# Nrf2 Regulates Basal Glutathione Production in Astrocytes

**DOI:** 10.3390/ijms26020687

**Published:** 2025-01-15

**Authors:** Jiali He, Sandra J. Hewett

**Affiliations:** Program in Neuroscience, Department of Biology, Syracuse University, Syracuse, NY 13210, USA; jiali_he@urmc.rochester.edu

**Keywords:** astrocytes, glutathione, Nrf2, glutamate cysteine ligase, system x_c_^−^, oxidative stress

## Abstract

Astrocytes produce and export glutathione (GSH), an important thiol antioxidant essential for protecting neural cells from oxidative stress and maintaining optimal brain health. While it has been established that oxidative stress increases GSH production in astrocytes, with Nrf2 acting as a critical transcription factor regulating key components of the GSH synthetic pathway, the role of Nrf2 in controlling constitutive GSH synthetic and release mechanisms remains incompletely investigated. Our data show that naïve primary mouse astrocytes cultured from the cerebral cortices of Nrf2 knockout (Nrf2^−/−^) pups have significantly less intracellular and extracellular GSH levels when compared to astrocytes cultured from Nrf2 wild-type (Nrf2^+/+^) pups. Key components of the GSH synthetic pathway, including xCT (the substrate-specific light chain of the substrate-importing transporter, system x_c_^−^), glutamate-cysteine ligase [catalytic (GCLc) and modifying (GCLm) subunits], were affected. To wit: qRT-PCR analysis demonstrates that naïve Nrf2^−/−^ astrocytes have significantly lower basal mRNA levels of xCT and both GCL subunits compared to naïve Nrf2^+/+^ astrocytes. No change in mRNA levels of glutathione synthetase (GS) or the GSH exporting transporter, Mrp1, was found. Western blot analysis reveals reduced protein levels of both subunits of GCL, while (seleno)cystine uptake into Nrf2^−/−^ astrocytes was reduced compared to Nrf2^+/+^ astrocytes, confirming decreased system x_c_^−^ activity. These findings suggest that Nrf2 regulates the basal production of GSH in astrocytes through constitutive transcriptional regulation of GCL and xCT.

## 1. Introduction

Astrocytes are the most abundant glial cell type in the central nervous system (CNS) [1]. They undertake important functions both during development and throughout adulthood [1]. During neuronal development, astrocytes are crucial for synaptogenesis [2]. In adults, astrocytes also exert a vital role in synaptic transmission [3], maintain an optimal extracellular environment [4,5], provide metabolic support [6], and contribute greatly to antioxidant defense [7]. Astrocytes exhibit greater resilience towards oxidative stress than neurons [8,9]. This superior antioxidant capacity of astrocytes can be largely attributed to the high levels of their intracellular antioxidants. Astrocytes are rich in ascorbate (vitamin C) [10], superoxide dismutase (SOD) [11], catalase (CAT) [12], and glutathione (GSH) [13]. In fact, they are the most abundant source of glutathione in the CNS [13], with levels being higher in astrocytes than neurons under physiological conditions both in vivo [14] and in vitro [15].

The biosynthesis of GSH in astrocytes and other cells is a tightly regulated process, ensuring its availability for various cellular functions. GSH synthesis involves two ATP-dependent enzymatic steps occurring in the cytosol [16,17]. First, glutamate and cysteine are conjugated by glutamate cysteine ligase (GCL), forming γ-glutamylcysteine. This first step is rate-limiting and is regulated by cysteine availability [18,19], transcriptional control of GCL subunits (GCLc and GCLm) [20], and holoenzyme formation [21,22]. In astrocytes, the availability of cysteine relies on system x_c_^−^, which imports cystine that will be rapidly reduced to cysteine intracellularly by thioredoxin reductase or glutathione reductase [23]. System x_c_^−^ activity is mainly regulated by the expression level of its substrate-specific subunit, xCT [23]. Next, glycine is added to the dipeptide by glutathione synthetase (GS), completing GSH synthesis. The second step is generally not regarded as rate-limiting since γ-glutamylcysteine levels are low due to their high turnover rate [24]. Finally, GSH is transported from astrocytes to the extracellular space predominantly via the multidrug resistance protein 1 (Mrp1) [25].

The transcription factor Nrf2 controls the expression of an array of genes associated with antioxidant defense, including those associated with GSH synthesis and release (reviewed in [26]). During oxidative stress, disruption of the Nrf2-Keap1 interaction results in Nrf2 activation [27]. This activation enhances the transcription of antioxidant genes [26,28], including the key GSH synthetic enzymes (GCLc, GCLm, GS), as well as transporters that regulate substrate import (system x_c_^−^) and GSH release (Mrp1) [29,30,31].

Nrf2 also exists at basal levels, albeit with a relatively short half-life of 20 min, and can translocate to the nucleus to drive gene transcription under normal physiological conditions [32,33]. This active basal level of Nrf2 regulates GSH production under physiological conditions in several tissues [31,32,34,35]. In the CNS, a study from Jeff Johnson’s lab demonstrated that naive Nrf2 knockout astrocytes have decreased basal intracellular GSH levels compared to wild-type astrocytes, potentially due to reduced basal GCLc and GCLm expression levels [36]. Subsequently, the same group examined primary neuronal cultures and found a similar decrease in intracellular GSH levels in Nrf2^−/−^ neurons [37]. However, unlike astrocytes, the reduction in neuronal GSH levels is solely accompanied by a decrease in the expression of GCLc [37]. A previous study reported reduced xCT protein levels in the meninges and cortex of Nrf2 null mice; however, the specific cell type(s) affected were not identified [38]. Astrocytes, which exhibit the highest levels of xCT mRNA [39,40], protein [41], and system x_c_^−^ [42] activity in the brain, are a likely candidate. Furthermore, prior research has not systematically compared mRNA, protein, and activity levels of key components involved in GSH synthesis and release across different Nrf2 genotypes in astrocytes. To address these knowledge gaps, our study systematically compares these parameters for system x_c_^−^, both GSH synthetic enzymes, and Mrp1 in naïve primary cortical astrocytes cultured from pups that were homozygous, heterozygous, or null for nuclear factor erythroid-derived 2-like 2 (*NFE2L2*), the gene encoding Nrf2.

## 2. Results

### 2.1. Effect of Loss of Nrf2 on Basal GSH Levels

**Figure 1:**
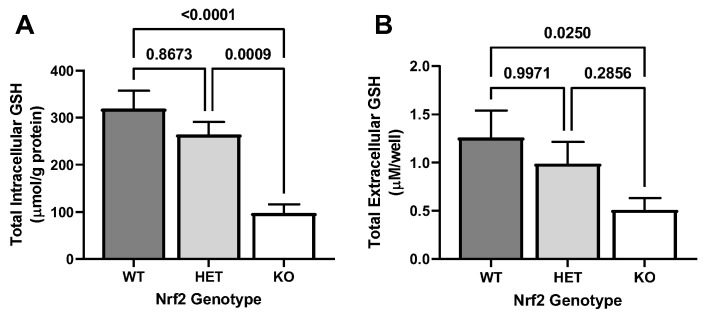
Impact of Nrf2 loss on constitutive GSH concentrations. Naïve primary cortical astrocyte cultures wild-type (WT), heterozygous (HET), and knockout (KO) for Nrf2 (*n* = 18 from five separate dissections) were incubated in serum-free medium for 48 h, after which total (**A**) intracellular and (**B**) extracellular GSH levels were measured. Data are expressed as mean + SEM. Exact *p*-values are shown for each pairwise comparison as determined by the Kruskal-Wallis test followed by Dunn’s multiple comparisons test.

Building upon previous findings from the Johnson lab [36], our study confirmed a genotype-dependent reduction in intracellular GSH concentrations (Figure 1A). Specifically, naïve Nrf2 KO astrocytes have a significantly lower concentration of intracellular GSH compared to both WT and HET astrocytes, with no significant difference in levels measured between WT and HET astrocytes observed (Figure 1A). Furthermore, we observe a similar pattern of reduction in extracellular GSH levels (Figure 1B). Extracellular levels of GSH were significantly lower in KO astrocytes when compared to WT only, with no significant difference observed between WT and HET cells or KO and HET cells observed (Figure 1B). Together, these data demonstrate a reduction in both intracellular and extracellular GSH levels in Nrf2 KO astrocytes.

### 2.2. Effect of Loss of Nrf2 on Constitutive xCT Expression Levels and Function

**Figure 2:**
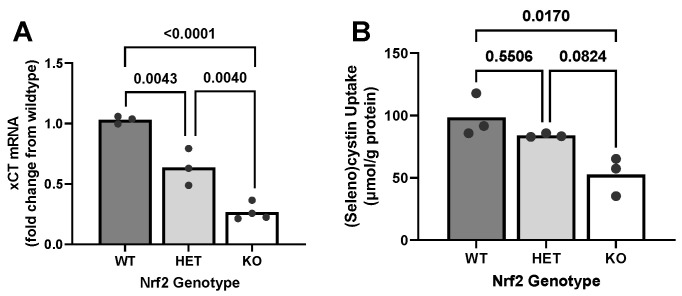
The impact of Nrf2 loss on constitutive xCT mRNA levels and system x_c_^−^ activity. (**A**) xCT mRNA (*n* = 3–4 from three separate dissections) from primary cortical astrocyte cultures wild-type (WT), heterozygous (HET), or knockout (KO) for Nrf2 were assessed via RT-qPCR. The horizontal bar represents the mean xCT mRNA levels expressed as fold change from wild-type of the independent replicates (black circles) (**B**). Astrocytes of different Nrf2 genotypes (*n* = 3 from three different dissections) were incubated for 30 min with 25 µM selenocystine, after which intracellular selenocystine concentrations were determined. The horizontal bar represents the mean selenocystine uptake (normalized to protein content) of the independent replicates (black circles). Exact *p*-values are shown for each pairwise comparison as determined by one-way ANOVA followed by Šídák’s multiple comparisons test.

Given the abundance of glutamate and glycine in astrocytes [43], cysteine is the rate-limiting substrate for GSH synthesis, as is common in many cells [18,44]. Cysteine is derived from cystine that is imported into the cell by the transporter system x_c_^−^ via its subunit xCT [45,46]. Thus, we investigated the regulatory role of Nrf2 on constitutive xCT expression (Figure 2A) and system x_c_^−^ activity (Figure 2B). xCT mRNA levels reflected the Nrf2 genotype of the astrocyte culture: WT astrocytes had the highest levels, HET cells had intermediate levels, and KO astrocytes had the lowest xCT mRNA levels (Figure 2A). Despite the significant reduction in xCT mRNA in HET cells, there was no significant difference in system x_c_^−^ activity between Nrf2 WT and HET cells. However, activity in KO cells was significantly lower when compared to WT (Figure 2B). Overall, Nrf2 KO astrocytes display significantly reduced xCT mRNA levels and decreased cystine uptake.

### 2.3. Effect of Loss of Nrf2 on Constitutive GCL Expression Levels

**Figure 3:**
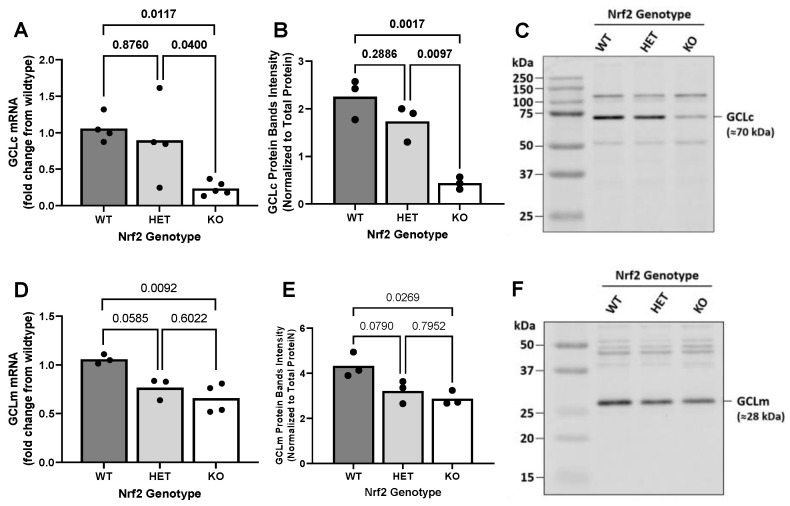
The impact of Nrf2 loss on constitutive glutamate cysteine ligase (catalytic and modifier subunits) mRNA and protein levels. (**A**) GCLc mRNA (*n* = 4–5 from four different dissections), (**B**) GCLc protein (*n* = 3 from three separate dissections), (**D**) GCLm mRNA (*n* = 3–4 from three different dissections), and (**E**) GCLm protein (*n* = 3 from three separate dissections) from primary cortical astrocyte cultures wild-type (WT), heterozygous (HET), or knockout (KO) for Nrf2 were measured as described in methods. The horizontal bar represents the mean expression levels of individual replicates, represented in black circles. Exact *p*-values are shown for each pairwise comparison as determined by ordinary one-way ANOVA followed by Šídák’s multiple comparisons test (**C**,**F**). Representative Western Blot detecting GCLc and GCLm, respectively.

To determine the impact of loss of Nrf2 on the first committed step in GSH synthesis, we evaluated the basal expression of GCLc and GCLm mRNA and protein in primary astrocytes cultured from mice that were WT, HET, or null (KO) for Nrf2 (Figure 3). Compared to both WT and HET astrocytes, KO astrocytes show markedly reduced levels of GCLc mRNA (Figure 3A) and GCLc protein (Figure 3B,C). Neither GCLc mRNA (Figure 3A) nor protein levels (Figure 3B,C) in HET astrocytes differed statistically from WT cells. Compared to WT astrocytes, Nrf2 KO astrocytes show consistently lower levels of both GCLm mRNA (Figure 3D) and GCLm protein (Figure 3E,F). Astrocytes heterozygous for Nrf2 displayed a phenotype closely resembling KO cells, although the reduction from WT cells did not reach statistical significance (Figure 3D–F). In summary, astrocytes deficient in Nrf2 have significantly reduced mRNA and protein expression of both GCL subunits.

### 2.4. Effect of Loss of Nrf2 on Constitutive GS Expression Levels

**Figure 4:**
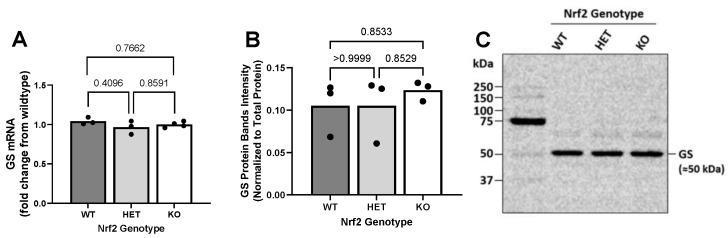
Loss of Nrf2 does not affect constitutive glutathione synthetase (GS) mRNA and protein levels. (**A**) GS mRNA (*n* = 3–4 from three different dissections), and (**B**) protein (*n* = 3 from three separate dissections) from primary cortical astrocyte cultures wild-type (WT), heterozygous (HET), and knockout (KO) for Nrf2 were measured as described in methods. The horizontal bar represents the mean expression levels of individual replicates, represented by the black circles. Exact *p*-values are shown for each pairwise comparison as determined by ordinary one-way ANOVA followed by Šídák’s multiple comparisons test. (**C**) Representative Western Blot.

To determine whether loss of Nrf2 may affect the second step in GSH synthesis, we compared the mRNA and protein levels of GS. Interestingly, neither the reduction (HET) nor absence (KO) of Nrf2 resulted in any change in the levels of basal GS mRNA (Figure 4A) or protein (Figure 4B,C).

### 2.5. Effect of Loss of Nrf2 on Constitutive Mrp1 Expression Levels

**Figure 5:**
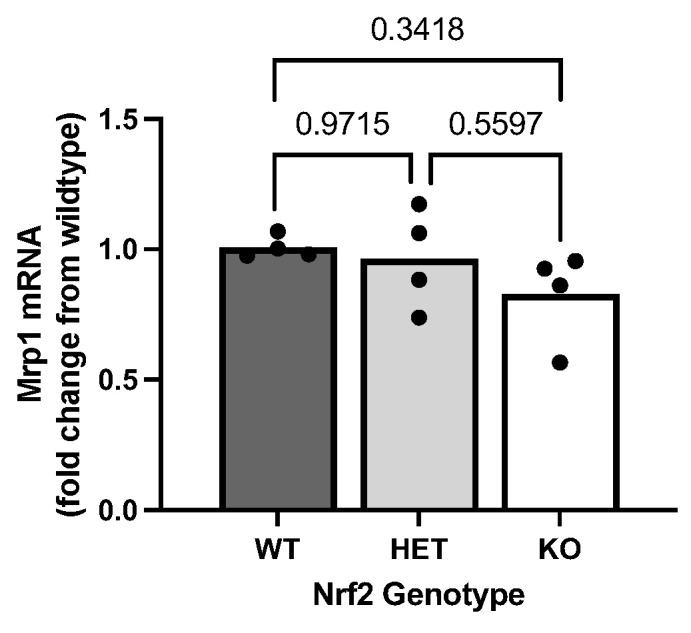
Loss of Nrf2 does not affect constitutive multidrug resistance protein 1 (Mrp1) mRNA levels. Mrp1 mRNA (*n* = 3 from three different dissections) from primary cortical astrocyte cultures wild-type (WT), heterozygous (HET), and knockout (KO) for Nrf2 were measured via RT-qPCR as described in methods. The horizontal bar represents the mean expression levels of individual replicates (black circles). Exact *p*-values are shown for each pairwise comparison as determined by ordinary one-way ANOVA followed by Šídák’s multiple comparisons test.

To assess the impact of Nrf2 deficiency on the primary GSH-exporting transporter, we quantified the basal expression level of Mrp1 mRNA. Our findings demonstrate that reduction or absence of Nrf2 did not result in any significant change in Mrp1 mRNA (Figure 5) levels. Protein levels were not assessed as we were unable to validate commercial Mrp1 antibodies.

## 3. Discussion

Under basal conditions, constitutive transcription factors (CTFs) regulate genes that contribute to the maintenance of cellular homeostasis. These CTFs include Sp1 [47], CREB, CREM, ATF-1, and ATF-2 [48]. While Nrf2 has been well recognized as an inducible transcription factor, emerging evidence suggests that a fraction of Nrf2 constitutively localizes to the nucleus, where it continuously activates ARE-driven antioxidant gene expression [32], thereby constitutively regulating genes involved in antioxidant defense. This constitutive activity has been demonstrated in liver [34], spleen [35], hepatic cell lines [32], fibroblasts [31], as well as in neurons [37] and astrocytes [36]. In the liver, Nrf2 protein levels were found to be depleted in the nuclei of Nrf2 KO mice, together with reduced GSH levels and reduced expression of ARE-driven genes [34]. Nrf2 KO mice also exhibited decreased expression levels of ARE-driven genes in the spleen and in fibroblasts [31,35]. ChIP assay revealed constitutive binding of Nrf2 to the ARE element in the nucleus in a hepatic cell line [32]. With relevance to this study, primary astrocytes and neurons cultured from Nrf2 KO mice contain significantly lower intracellular GSH levels, along with reduced basal expression of GCLc and GCLm mRNA and protein, compared to cultures derived from Nrf2 wild-type mice [36,37]. However, the full scope of Nrf2’s regulation of the GSH synthetic and release mechanisms—including system x_c_^−^, both GSH synthetic enzymes, and Mrp1—in astrocytes remains incompletely explored, making it the focus of this study.

In agreement with previous findings from the Johnson lab, which reported a 48% reduction in intracellular GSH in Nrf2^−/−^ astrocytes [22], we observed a 69% reduction in intracellular GSH level in Nrf2^−/−^ astrocytes compared to Nrf2^+/+^ astrocytes. Together these findings reveal that intracellular GSH content is not fully depleted when Nrf2 is knocked out, suggesting that other mechanisms contribute to constitutive GSH production in astrocytes. One potential regulatory pathway is NFκB [49,50]. Whether NFκB contributes to the maintenance of constitutive GSH levels in astrocytes remains to be elucidated.

Extending our results and the findings from the Johnson lab, we also found decreased extracellular GSH in the cell culture media of Nrf2^−/−^ astrocytes compared to Nrf2^+/+^ astrocytes. Given that astrocytes release 10% of their total GSH per hour into the extracellular space [51], it’s not surprising that the 59% reduction in extracellular GSH levels closely matches the reduction observed intracellularly. This correlation between extracellular and intracellular reductions implies that the GSH exporting mechanism remains robust in Nrf2 KO astrocytes. This idea is supported by our finding that mRNA levels of Mrp1, the gene that encodes for the protein that mediates 60% of GSH release in astrocytes [25], remain unchanged across Nrf2 genotypes. Because we were not able to validate any Mrp1 antibody for Western blot, further studies are needed to confirm the stability of Mrp1 protein levels.

These findings contrast with studies in embryonic mouse fibroblasts, where knockout of Nrf2 results in a significant reduction in both Mrp1 mRNA and protein levels compared to wild-type cells [31], a finding further corroborated by identifying Mrp1 (*Abcc1*) in a list of 645 basal gene targets of Nrf2 in mouse embryonic fibroblasts [52]. The different results between primary astrocytes and embryonic fibroblasts suggest that Nrf2 may regulate Mrp1 differently across cell types and/or perhaps developmental stages. With respect to the latter, the regulatory function of Nrf2 might be affected by the presence of other nuclear proteins during maturation. For instance, the nuclear enzyme TOP2A (Type IIA topoisomerase) plays essential roles in transcription by catalyzing transient DNA double strand breaks. TOP2A was assessed by RNA-seq and qRT-PCR to be highly expressed in immature astrocytes but was nearly undetectable in mature astrocytes, both in purified human astrocytes in vivo and in cultured rat astrocytes in vitro [53]. Thus, TOP2A might mediate Mrp1 expression during development.

Other potential release mechanisms for GSH that were not assessed in this study include gap junction hemichannels [54,55] and organic anion transporting polypeptide (OATP) [56,57]. Previous studies have shown that the activation of Nrf2 downregulates both hemichannels and OATP [58,59]. Thus, the absence of Nrf2 could potentially lead to an upregulation of these channels, which would be inconsistent with the observed reduction in extracellular GSH concentration observed. Therefore, it is unlikely that these pathways contribute to the decreased extracellular GSH levels observed in our Nrf2 knockout astrocyte cultures.

Cystine uptake has been reported to be rate-limiting for GSH synthesis in primary astrocyte cultures [60]. Interestingly, we find a 74% reduction in mRNA levels of xCT, the substrate-specific light chain of system x_c_^−^—which closely correlates with the decrease in intracellular GSH produced (69%). Although we attempted to quantify xCT protein levels, it is well known that xCT antibodies are unreliable [61], and our attempts to validate commercially available antibodies were unsuccessful. Instead, we measured (seleno)cystine uptake into our astrocytes to determine system x_c_^−^ activity [62]. Astrocytes derived from Nrf2 KO mice demonstrated a 47% reduction in uptake. This differs from our mRNA results but is in keeping with a study reporting a 44% reduction in xCT protein levels in cortical tissue of Nrf2 KO mice [38]. This consistency between reductions in cortical tissue [38] and our primary astrocyte cultures supports existing evidence that astrocytes predominately contribute to system x_c_^−^ expression in the cortex. Specifically, previous studies have shown that astrocytes express the highest levels of xCT [39,40,41] and exhibit the highest system x_c_^−^ activity [42] compared to other brain cell types.

We find the discrepancy between reductions in system x_c_^−^ activity (47%) and xCT mRNA level (74%) in Nrf2 KO astrocytes not too surprising considering previous studies reporting that mRNA abundance only explains 20–40% of the protein variability in mammalian cells [63,64] and in bacteria [65]. Other factors contributing to protein abundance include RNA and protein stability [65]. Our lab previously reported the xCT mRNA half-life in astrocytes under basal conditions to be 4 ± 1.9 h [66], which is relatively shorter than the reported median mRNA half-life of 7.1 h calculated from nearly 20,000 different genes in mouse embryonic stem cells [67]. Hence, the instability of xCT mRNA likely accounts for part of the decreased turnover rate from mRNA to functional protein. Another major factor is cytosol-localized xCT protein, which can function as a transporter only on the plasma membrane. Although we were not able to measure the relative amount of cytosolic and membrane xCT protein levels, detectable amounts of intracellular xCT proteins have been reported in mouse glial [68] and liver cells [69]. These cytosolic xCT proteins could be trafficked to the cell surface to form a functional transporter [70,71]. Thus, the smaller reduction in system x_c_^−^ activity as compared to mRNA levels could be accounted for by mRNA instability and xCT protein trafficking.

The activity of system x_c_^−^ influences cellular GSH levels under various contexts. In astrocytes, our lab found that enhancing xCT expression with IL-1β [72] or sodium arsenite (unpublished data) increased GSH production and release into the culture media. Conversely, treating astrocytes with system x_c_^−^ inhibitors such as erastin [73], sulfasalazine [74], or 4-CPG (our unpublished data) significantly decreased GSH levels. Additionally, extracellular GSH levels were reduced in astrocytes derived from mice lacking system x_c_^−^ [66]. Therefore, the decreased constitutive activity of system x_c_^−^ in Nrf2 KO astrocytes likely contributes to the observed reduction in GSH.

While it is possible that the reduction in substrate availability alone could explain the decrease in GSH observed in Nrf2 KO astrocytes, reductions in GCL activity could also contribute. We found a 78% reduction in mRNA of GCLc and a 38% reduction in mRNA levels of GCLm. GCLm modifies GCL function by lowering the Michaelis-Menten constant (Km) of GCLc for glutamate and ATP and raising the inhibitory constant (Ki) of GSH on GCLc inhibition [75,76]. Hence, overall, GCLc activity can be enhanced by the presence of GCLm. Demonstrating its importance, kidney, pancreas, and plasma from GCLm knockout mice contain just 10–40% of GSH compared to the same tissues or plasma from wild-type mice [77]. Additionally, pharmacological reductions in GCL activity have been demonstrated to lower liver, kidney, and brain tissue GSH levels [63], and genetic deletion of one allele of GCLc ^(+/−)^ decreased liver GSH concentration by 20% [78]. Notably, the reductions in GCLc and GCLm proteins in Nrf2 KO astrocytes closely mirrored the mRNA results, with levels being reduced by 80% and 29%, respectively. This is in general agreement with what Johnson’s lab reported previously in Nrf2 KO astrocytes—54% reduction in GCLc mRNA and 15% reduction in GCLm mRNA [36]. While these changes are not as large as our qPCR results, this may simply have to do with the differing dynamic ranges of qPCR and microarray, with qPCR generally exhibiting better sensitivity [79]. Finally, we found no change in either mRNA or protein levels for GS in Nrf2 KO astrocytes.

A limitation of this study is that all experiments are performed in cultured astrocytes, which lack Nrf2 from the beginning of development. The properties of cells or tissue samples from Nrf2 KOs might differ from samples where Nrf2 is transiently reduced using antisense, siRNA, or Nrf2-inhibiting drugs. Indeed, phenotypic differences between knockout and knockdown systems have been reported in multiple model organisms, including drosophila [80], zebrafish [81], and mice [82]. This distinction is crucial as it elucidates the potential disparities between the long-term absence of Nrf2 and its transient reduction. These discrepancies could originate from non-specific binding of the antisense or siRNA reagents used in knockdown treatments or result from genetic compensation in the knockout animals [83]. Additionally, the use of cultured astrocytes may not fully replicate the complex in vivo environment, where cell-cell interactions play significant roles in cellular function. This limitation suggests that findings from cultured astrocytes should be interpreted with caution when extrapolating to in vivo conditions.

**Figure 6:**
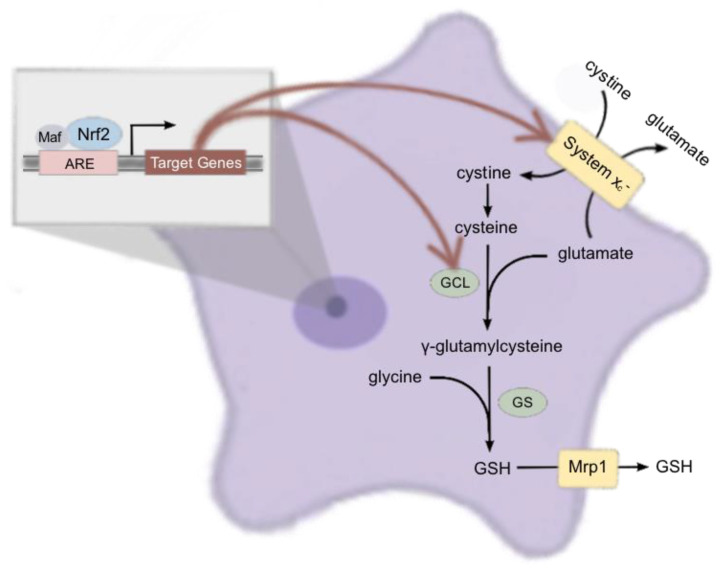
Nrf2 regulates basal GSH production in astrocytes through constitutive regulation of xCT and GCL.

In conclusion, our results demonstrate that naïve primary astrocytes, cultured from cerebral cortices of neonatal pups null for Nrf2, had significantly lower levels of intracellular GSH. This reduction was mirrored by a decrease in extracellular GSH and correlated with decreased xCT mRNA levels and functional activity of the system x_c_^−^ transporter. Moreover, we observed significant reductions in mRNA and protein levels of GCLc and GCLm, key enzymes involved in GSH synthesis, while the expression of GS and Mrp1 remained unaffected. All of the genes encoding for the proteins assessed —xCT [84], GCLc [85], GCLm [86], GS [87], and Mrp1 [88]—have the ARE element on their promoter region (Figure 6). Overall, our findings contribute to the growing body of evidence describing the indispensable role of Nrf2 in preserving physiological GSH levels within cortical astrocytes, thereby supporting the rationale for Nrf2-targeted drug development in neurological disorders [89,90,91], such as Parkinson’s Disease (PD), Alzheimer’s Disease (AD), Amyotrophic Lateral Sclerosis (ALS), Huntington’s Disease (HD), and Multiple Sclerosis (MS) (reviewed in [13,92]), where oxidative stress is a crucial mechanism leading to cellular injury [93].

## 4. Materials and Methods

### 4.1. Experimental Animals

This study was conducted in accordance with the National Institute of Health guidelines for the use of experimental animals and has been approved by the Institutional Animal Care and Use Committee of Syracuse University. Mice were maintained on a 12 h light/dark schedule and provided mouse chow and water ad libitum. Nrf2 null mutant male mice of at least 6 weeks of age (B6.129X1-Nfe2l2tm1Ywk/J; JAX stock #017009) were first bred with wild-type female mice (C57BL/6J; JAX stock #000664) 6–12 weeks of age to generate F1 Nrf2 heterozygous mice. F1 heterozygous breeding pairs were subsequently bred to obtain pups that are wild-type, heterozygous, and null for Nrf2. Female heterozygous mice were used for breeding starting at 6–8 weeks of age, continuing until approximately 8 months, or until they lost fecundity, defined as failing to produce litters after three breeding attempts. Neonatal male and female pups, postnatal 1–3 days, were used for astrocyte culture.

### 4.2. Experimental Media/Buffers

Cell culture media and experimental buffer compositions were as follows:

- Media stock (MS): L-glutamine-free modified Eagle’s medium (Earl’s salt; Corning, Corning, NY, USA) supplemented with L-glutamine, glucose, and sodium bicarbonate to a final concentration of 2.0, 25.7, and 28.2 mM, respectively. Phenol-red-free MS is made with Phenol-red-free EMEM (Quality Biological, Gaithersburg, MD, USA).
- Glial plating media: MS containing 10% heat-inactivated fetal bovine serum (FBS; Hyclone or VWR) and 10% heat-inactivated calf serum (CS; Hyclone or VWR), 20 ng/mL epidermal growth factor (Sigma-Aldrich, St. Louis, MO, USA), 50 IU penicillin, and 50 µg/mL streptomycin (Gibco/BRL, Waltham, MA, USA).
- Maintenance media-1: MS containing 10% CS and 50 IU penicillin/50 µg/mL streptomycin.
- Maintenance media-2: MS containing 3% CS and 50 IU penicillin/50 µg/mL streptomycin.

### 4.3. Single Pup Astrocyte Cultures

Nrf2 wild-type, heterozygous, and homozygous null mutant astrocytes were cultured from cerebral cortices of individual pups (postnatal 1–3 days) derived from Nrf2 heterozygous breeders as described [94]. In short, mouse brains were removed and maintained in magnesium- and calcium-free Hibernate^®^ E minus Calcium, minus Magnesium (BrainBits, Köln, Germany) for 5–7 h so that Nrf2 genotyping could be performed (https://www.jax.org/strain/017009; accessed on 11 October 2024). Dissociated cells of the same Nrf2 genotype (pooled cortices from 1–3 mice pups) were plated in multi-well plates (two wells of 6-well or twelve wells of 24-well, Corning Primaria) in glial plating media. Once confluent, monolayers were treated with 8 µM cytosine arabinoside (AraC; Sigma) once for 5–7 days to reduce the number of microglia. Cells were then placed in maintenance media-1 and fed once per week. One day prior to experimentation, cells were replaced with maintenance media-2 in preparation for serum-free conditions. Cultures were maintained at 37 °C in a humidified 5.0% CO_2_, 21% O_2_-containing incubator and were used at ≤35 days in vitro.

### 4.4. Measurement of GSH

Total extracellular and intracellular glutathione (GSH + GSSG) levels were determined using the luminescence-based GSH-Glo glutathione assay (Promega, Madison, WI, USA) per the manufacturer’s instructions. Cells were washed into maintenance media-2 the day before experimentation, after which they were put in serum-free and phenol-red-free MS and placed back in the incubator. After 48 h, cell culture media was collected for measurement of extracellular GSH ([GSH]_e_), and GSH-Glo reaction buffer was added directly to the astrocyte cultures to obtain supernatant for measurement of intracellular GSH ([GSH]_i_). These supernatants were diluted (1:5) using the GSH-Glo reaction buffer to keep relative light units within the dynamic range of the standards. To determine total GSH levels, glutathione disulfide (GSSG) within the samples was converted to GSH with the reducing agent TCEP-HCl (final concentration = 1 mM; 10 min; 25 °C; Sigma-Aldrich). While TCEP-HCl is not required for assessing intracellular GSH levels due to the reducing nature of the intracellular environment, it is essential for accurately measuring extracellular GSH levels, as the extracellular environment is oxidizing (Supplementary Figure S1). Luciferase activity was measured using a Synergy2 microplate reader (BioTek, Winooski, VT, USA). Total intracellular or extracellular GSH was calculated from standards prepared in GSH-Glo reaction buffer and phenol-red-free MS containing 2 mM L-glutamine, respectively. [GSH]_i_ was normalized to cellular protein.

### 4.5. Real-Time qPCR

RNA was isolated and first-strand cDNA synthesized as previously described [95]. RT-qPCR was performed using mouse-specific primer pairs [Taqman Gene Expression Assays, Applied Biosystems (Waltham, MA, USA): system x_c_^−^ light chain (xCT) (Mm00442530_m1), glyceraldehyde-3-phosphate dehydrogenase (Gapdh) (Mm99999915_g1), glutamate-cysteine ligase, catalytic subunit (GCLc) (Mm00802655_m1), glutamate-cysteine ligase, modifier subunit (GCLm) (Mm00514996_m1), glutathione synthetase (GS) (Mm00515065_m1), Multidrug resistance protein 1 (Mrp1/Abcc1) (Mm00456156_m1), hypoxanthine guanine phosphoribosyl transferase (Hprt) (Mm01545399_m1)] per manufacturer’s instructions. Reactions were run in triplicate in a Mastercycler^®^ RealPlex2 from Eppendorf (Hamburg, Germany), and relative quantification was performed on mean values using the comparative cycle threshold method (∆∆Ct), where Ct values of the transcript of interest were normalized to Gapdh (for xCT, GCLm, and GS) or Hprt (for GCLc and Mrp1). Ct values from the same sample were then compared to a calibrator Ct value (untreated cells) to determine the relative fold increase in mRNA. Gapdh and Hprt Ct values were confirmed to be unaffected by loss of Nrf2. Primer efficiencies between transcripts of interest and housekeeping genes did not differ by more than 5% (Supplementary Figure S2).

### 4.6. Immunoblotting

Protein expression was determined by Western blot analysis. Astrocytes in 6-well plates were washed twice with 1X HBSS (Corning Cellgro), followed by the addition of 1.6 mL of 0.05% Trypsin-EDTA (Gibco) for 5–10 min at 37 °C to facilitate detachment. To stop trypsin digestion, dislodged cells were transferred to an Eppendorf tube containing 1.6 mL maintenance media-1 followed by centrifugation at 300× *g* for 5 min at 4 °C. The pellet was resuspended in 3 mL PBS and respun at 300× *g* for 5 min at 4 °C. Pellets from two wells were combined and resuspended in 200 µL RIPA lysis buffer containing 50 mM Tris, 1% NP-40, 1% SDS, 0.15 M NaCl, 12 mM deoxycholic acid, 5 mM Iodoacetamide, 5 mM EDTA, and 1X Complete Protease Inhibitor (Roche, Basel, Switzerland), followed by incubation on ice for 30 min. Lysates were spun (2500× *g*; 5 min; 4 °C), and the protein concentration in the resulting supernatant was measured using the BCA assay (Pierce, Appleton, WI, USA). Ten µg of protein was resuspended in 4X SDS loading buffer (100 mM Tris, pH 6.8, 40% glycerol, 2% SDS, 50 mM EDTA, 6% β-mercaptoethanol, 0.08% bromophenol blue) and then boiled for 5 min to denature proteins. Proteins were separated by SDS-PAGE under reducing conditions and then electrophoretically transferred to a polyvinylidene difluoride (PVDF) membrane (0.45 µm, Immobilon). Each membrane was stained with LI-COR Revert^TM^ 700 so that the protein loading in each lane could be precisely quantified for subsequent normalization. After destaining, membranes were blocked in Odyssey blocking buffer (LI-COR, Lincoln, NE, USA) for 1 h at room temperature followed by overnight incubation (4 °C) with the following primary antibodies: GCLc (0.93 µg/mL; rabbit polyclonal; ABclonal, Woburn, MA, USA), GCLm (0.38 µg/mL; rabbit polyclonal; ABclonal), GS (0.51 µg/mL; mouse monoclonal; Novus Biologicals). Proteins of interest were detected using species-specific secondary antibodies labeled with spectrally distinct IRDye^®^ fluorescent dyes and visualized using the LI-COR ODYSSEY^®^ Fc imaging system (LI-COR). Analysis was performed using Image Studio 3.1 (LI-COR). The antibody-antigen interaction has been verified to be within the linear range, ensuring the amount of protein and antibody concentration tested is appropriate. Protein levels were normalized to values obtained with the total protein stain to account for any variations in sample loading and transfer efficiency (Supplementary Figure S3).

### 4.7. (Seleno)Cystine Uptake Assay

System x_c_^−^ activity was estimated by measuring the uptake of selenocystine as described [62]. Cells were switched into maintenance media-2 the day before experimentation. On the day of experimentation, culture media was replaced by thorough exchange (3×; 1.6 mL) with an amino acid-free balanced salt solution (BSS) containing 116 mm NaCl, 5.4 mm KCl, 0.8 mm MgSO_4_, 1 mm NaH_2_PO_4_, 26.2 mm NaHCO_3_, 1.8 mm CaCl_2_, and 10 mM glucose. After 5 min, the BSS was aspirated completely and replaced with 1.6 mL 25 µM Selenocystine (Sigma-Aldrich) diluted in BSS. Then cells were incubated in a 37 °C CO_2_ incubator for a duration of 30 min, after which they were thoroughly washed with PBS (3×, 3 mL). The PBS was aspirated, and 250 µL of cold methanol was added to induce permeabilization, facilitating the release of selenocystine. Methanol supernatants were diluted 1:1 in H_2_O, then incubated with 100 mM MES (2-ethanesulfonic acid) buffer (pH 6.0) containing 10 µM FOdA (Sigma-Aldrich) and 200 µM TCEP (Sigma-Aldrich) at 37 °C for 30 min in a 96-well black plate (Thermo Scientific™ Nunc™, Waltham, MA, USA). The reaction between selenocysteine and fluorescein O, O’-diacrylate (FOdA) results in the generation of fluorescein, whose fluorescence intensity was measured with an excitation wavelength of 485 nm and an emission wavelength of 535 nm using a Synergy2 microplate reader (BioTek). Selenocystine concentrations were determined based on selenocystine standards that were prepared in 50% methanol and 50% H_2_O. Cellular protein was then extracted by RIPA buffer (50 mM Tris, 1% NP-40, 1% SDS, 0.15 M NaCl, 12 mM deoxycholic acid) and quantified by BCA assay kit (Pierce). The selenocystine concentration in each culture was normalized to the protein content of the respective wells to account for variations in cell quantity. The specificity of the (seleno)cystine uptake assay for system x_c_^−^ has been validated using a system x_c_^−^ inducer and inhibitor (Supplementary Figure S4).

### 4.8. Statistical Analysis

All statistical analyses were performed using GraphPad Prism Version 9.4.1 as specified in each figure legend. To assess normality, the Shapiro-Wilk test was performed. Non-normally distributed data were analyzed by non-parametric tests or were transformed prior to analysis. Geometric means were used for mRNA statistical analysis via one-way ANOVA. GSH data was analyzed by the Kruskal-Wallis test. Protein data were analyzed by one-way ANOVA blocked by experiment day. Exact *p*-values are provided on each graph.

## Data Availability

Data are contained within the article.

## Supplementary Materials

The following supporting information can be downloaded at: https://www.mdpi.com/article/10.3390/ijms26020687/s1.
