# Supplementary material for: Nrf2 Regulates Basal Glutathione Production in Astrocytes
Source: Int J Mol Sci. 2025 Jan 15;26(2):687. doi: 10.3390/ijms26020687 (PMC11765531; doi:10.3390/ijms26020687)
Supplement: Supplementary file 1 [file ijms-26-00687-s001.zip › ijms-3337579-supplementary.pdf]

Supplemental Figures

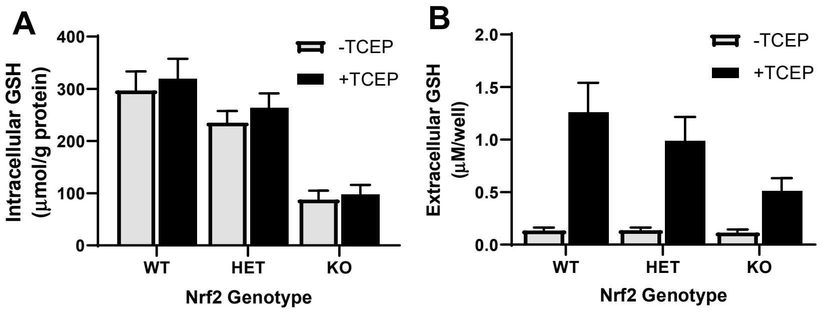

Supplementary Figure S1. Effect of TCEP-HCl on intracellular and extracellular GSH levels. Primary cortical astrocyte cultures wildtype (WT), heterozygous (HET), and knockout (KO) for Nrf2 (n=18 from five dissections) were incubated in serum-free medium for 48 h, after which total **(A)** intracellular and **(B)** extracellular GSH levels were measured in the absence or presence of the reducing agent TCEP-HCl. Data are expressed as mean + SEM.

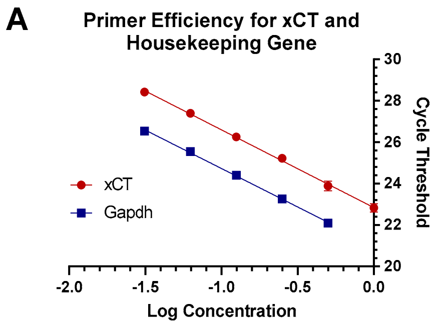

qPCR Primer Target	Simple Linear Regression Equation	R squared value	Primer Efficiency $(10^{\frac{-1}{slope}} - 1) * 100\%$
xCT	Y = -3.752*X + 22.84	R^2 = 0.9956	84.75%
Gapdh	Y = -3.711*X + 21.02	R^2 = 0.9983	85.98%

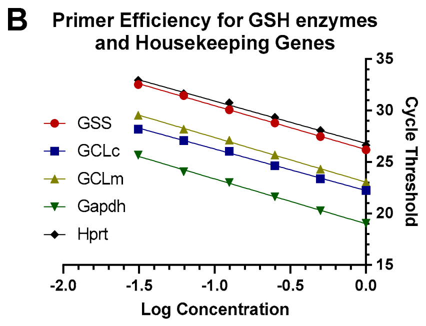

qPCR Primer Target	Simple Linear Regression Equation	R squared value	Primer Efficiency $(10^{\frac{-1}{slope}} - 1) * 100\%$
GSS	Y = -4.256*X + 26.22	R^2 = 0.9969	71.78%
GCLc	Y = -4.036*X + 22.23	R^2 = 0.9975	76.92%
GCLm	Y = -4.336*X + 23.05	R^2 = 0.9976	70.07%
Gapdh	Y = -4.354*X + 19.01	R^2 = 0.9954	69.70%
Hprt	Y = -4.117*X + 26.81	R^2 = 0.9941	74.94%

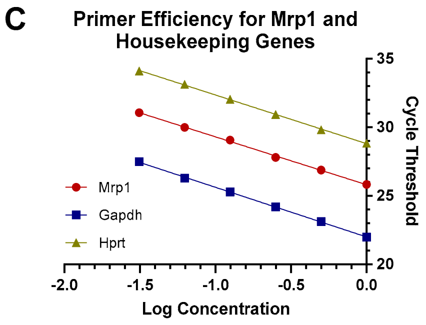

qPCR Primer Target	Simple Linear Regression Equation	R squared value	Primer Efficiency $(10^{\frac{-1}{slope}} - 1) * 100\%$
Mrp1	Y = -3.492*X + 25.82	R^2 = 0.9938	93.36%
Gapdh	Y = -3.621*X + 22.00	R^2 = 0.9993	88.87%
Hprt	Y = -3.549*X + 28.81	R^2 = 0.9986	91.32%

Supplementary Figure S2. RT-qPCR primer efficiencies. RT-qPCR primers targeting **(A)** xCT and Gapdh, **(B)** GSH synthetic enzymes (GCLc, GCLm, GS) and housekeeping genes (Gapdh and Hprt), and **(C)** Mrp1 and housekeeping genes (Gapdh and Hprt) were each tested on the same RT-qPCR plate with a serial diluted cDNA concentration gradient. Data are expressed as mean ±

SEM; primer efficiencies were calculated from the slope of each primer's simple linear regression equation.

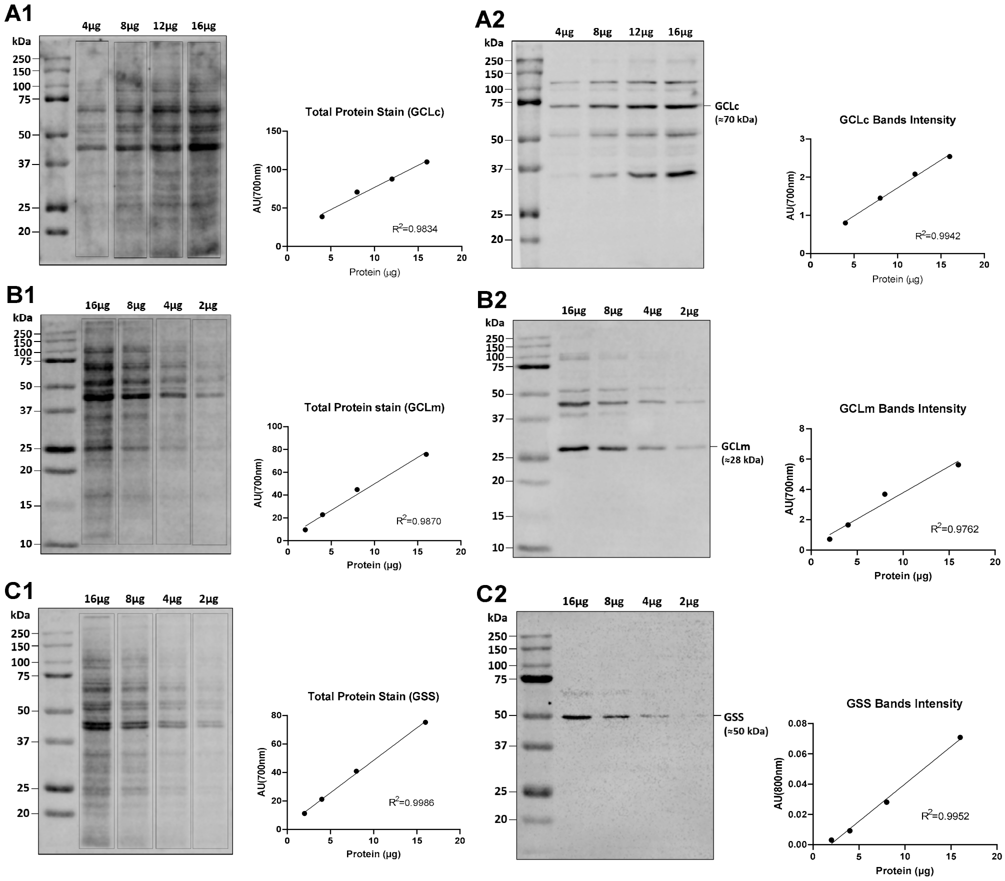

Supplementary Figure S3. Optimization of GSH synthetic enzyme antibodies. Dilution series of proteins harvested from primary cortical astrocyte cultures (GCLc: 4, 8, 12, 16 μg; GCLm and GS: 2, 4, 8, 16 μg) were separated under reducing conditions via SDS-PAGE (10% for GCLc and GS, 12% for GCLm). Accuracy of the serial dilution was confirmed using total protein levels visualized using RevertTM Total Protein Stain (LI-COR Biosciences). (A1,A2) GCLc, (B1,B2) GCLm, and (C1,C2) GS proteins were detected with two dilutions of antibody (1:500 and 1:1000) via Western blot analysis with representative blots shown. Data curves were fit via linear regression: (A1) total protein stain $R^2 = 0.9834$, (A2) GCLc protein bands $R^2 = 0.9942$, (B1) total protein stain $R^2 = 0.9870$, (B2) GCLm protein bands $R^2 = 0.9762$, (C1) total protein stain $R^2 = 0.9986$, (C2) GS protein bands $R^2 = 0.9952$. Optimal protein amounts (10 μg for GCLc, GCLm, and GS) were selected from the middle of the linear range. Final antibody concentrations chosen for experimentation are 0.928 μg/mL for GCLc, 0.375 μg/mL for GCLm, and 0.51 μg/mL for GS.

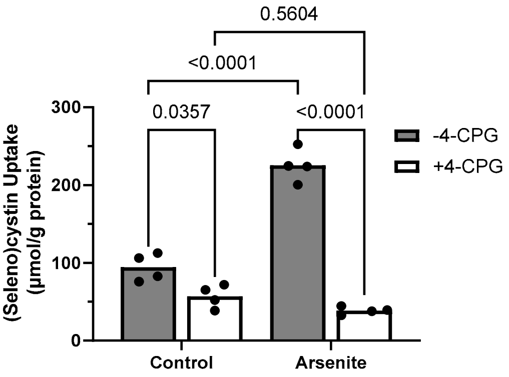

Supplementary Figure S4. The system x_c^- inhibitor 4-CPG blocks arsenite-mediated (seleno)cystine uptake increase. Primary cortical astrocyte cultures (n=4 from two separate dissections) were pre-treated with arsenite (15 µM) or its vehicle for 24 hours, after which cells were incubated for 30 min with 25 µM selenocystine in the presence or absence of 500 µM 4-CPG, and then intracellular selenocystine concentrations were determined as described in methods. The horizontal bar represents the mean selenocystine uptake (normalized to respective protein content) of the independent replicates (black circles). Group differences were determined by two-way ANOVA followed by Šídák's multiple comparisons.
